# Fine-Grained and Lightweight OSA Detection: A CRNN-Based Model for Precise Temporal Localization of Respiratory Events in Sleep Audio

**DOI:** 10.3390/diagnostics16040577

**Published:** 2026-02-14

**Authors:** Mengyu Xu, Yanru Li, Demin Han

**Affiliations:** 1 Department of Sleep Medical Center, Beijing Tongren Hospital, Capital Medical University, Beijing 100730, China; ent_xu@ccmu.edu.cn; 2 Key Laboratory of Otorhinolaryngology Head and Neck Surgery, Ministry of Education, Capital Medical University, Beijing 100730, China; 3 Xinjiang Key Laboratory of Biopharmaceuticals and Medical Devices, Xinjiang Medical University, Urumqi 830054, China

**Keywords:** obstructive sleep apnea, sleep audio analysis, deep learning, convolutional recurrent neural network, respiratory event detection

## Abstract

**Background:** Obstructive Sleep Apnea (OSA) is highly prevalent yet underdiagnosed due to the scarcity of Polysomnography (PSG) resources. Audio-based screening offers a scalable solution, but often lacks the granularity to precisely localize respiratory events or accurately estimate the Apnea-Hypopnea Index (AHI). This study aims to develop a fine-grained and lightweight detection framework for OSA screening, enabling precise respiratory event localization and AHI estimation using non-contact audio signals. **Methods:** A Dual-Stream Convolutional Recurrent Neural Network (CRNN), integrating Log Mel-spectrograms and energy profiles with BiLSTM, was proposed. The model was trained on the PSG-Audio dataset (Sismanoglio Hospital cohort, 286 subjects) and subjected to a comprehensive three-level evaluation: (1) frame-level classification performance; (2) event-level temporal localization precision, quantified by Intersection over Union (IoU) and onset/offset boundary errors; and (3) patient-level clinical utility, assessing AHI correlation, error margins, and screening performance across different severity thresholds. Generalization was rigorously validated on an independent external cohort from Beijing Tongren Hospital (60 subjects), which was specifically curated to ensure a relatively balanced distribution of disease severity. **Results:** On the internal test set, the model achieved a frame level macro F1 score of 0.64 and demonstrated accurate event localization, with an IoU of 0.82. In the external validation, the audio derived AHI showed a strong correlation with PSG-AHI (r = 0.96, MAE = 6.03 events/h). For screening, the model achieved sensitivities of 98.0%, 89.5%, and 89.3%, and specificities of 88.9%, 90.9%, and 100.0% at AHI thresholds of 5, 15, and 30 events per hour, respectively. **Conclusions:** The Fine-Grained and Lightweight Dual-Stream CRNN provides a robust, clinically interpretable solution for non-contact OSA screening. The favorable screening performance observed in the external cohort, characterized by high sensitivity for mild cases and high specificity for severe disease, highlights its potential as a reliable tool for accessible home-based screening.

## 1. Introduction

Obstructive sleep apnea (OSA) is a chronic disorder characterized by recurrent upper airway collapse during sleep, manifesting as snoring, intermittent apnea, and sleep fragmentation. These abnormalities are strongly associated with an increased risk of hypertension, stroke, coronary artery disease, and other severe comorbidities [1,2,3]. Currently, the clinical gold standard for diagnosing OSA is polysomnography (PSG). However, PSG involves overnight monitoring of physiological signals in specialized laboratories and requires time-consuming manual scoring by trained technicians [4,5].

In China, an estimated 176 million adults aged 30 to 69 are at high risk of OSA, representing the largest affected population globally [6]. Despite this prevalence, a 2023 survey indicates that there are only approximately 3000 sleep medicine centers nationwide, each with a limited daily capacity of fewer than five PSG tests. Consequently, the current diagnostic capability covers less than 20% of the population in need, creating a severe disparity between supply and demand [7]. To address this shortage, unattended portable monitoring (PM) has been adopted as an alternative to PSG for patients with a high pre-test probability of OSA and no significant comorbidities [8]. However, its scalability remains limited by the requirement for professional manual scoring and the slow turnover rate of devices. In recent years, alternative screening approaches have been explored to overcome these barriers, including artificial intelligence models based on clinical-demographic data [9] and wearable devices equipped with single- or multi-channel smart sensors [10]. Furthermore, emerging non-contact sensing solutions, such as smart mattresses [11] and millimeter-wave radar [12], have also shown potential for OSA screening. Despite these advancements, these methods often demonstrate variable performance stability or rely heavily on specific hardware. Consequently, they still face logistical challenges that hinder large-scale deployment. Given these constraints, there is an urgent imperative to develop a convenient, accurate, and scalable screening method.

Sleep audio analysis offers a promising solution to this challenge. Specifically, snoring, as the most prominent symptom of OSA and the primary complaint prompting clinical consultation, contains acoustic characteristics that reflect upper airway obstruction and correlate with OSA-related complications [13,14]. Notably, unlike PSG and other screening approaches, the acquisition of sleep audio is non-invasive and does not rely on specialized medical instrumentation. High-quality audio can be captured using consumer-grade devices such as smartphones, making this approach highly suitable for widespread screening in community settings.

In recent years, the integration of Artificial Intelligence has advanced audio-based OSA screening, marking a transition from manual feature engineering to end-to-end deep learning. Early approaches primarily relied on handcrafted acoustic features, including Mel-frequency Cepstral Coefficients, Linear Prediction Coefficients, spectral flux, pitch, zero-crossing rate, and spectral entropy. These features were combined with traditional machine learning algorithms such as Support Vector Machines to classify sleep sounds [15,16,17,18]. However, these methods often exhibited limited robustness against environmental noise. Furthermore, they typically required complex manual feature selection processes dependent on domain expertise, which limited their generalizability. The adoption of Deep Learning shifted the paradigm toward automated feature extraction. Convolutional Neural Networks became a standard method for learning time-frequency representations directly from spectrograms, often integrated with Recurrent Neural Networks to capture the temporal evolution of respiratory patterns [19,20,21,22,23]. Although Transformer-based architectures have recently gained prominence in general audio tasks and are emerging in OSA screening [24,25], their quadratic computational complexity and substantial parameter count present challenges for resource-constrained edge deployment. Moreover, the global attention mechanism may be functionally redundant for the semi-periodic, physiological nature of breathing sounds. Consequently, Convolutional Neural Networks and Convolutional Recurrent Neural Networks remain practical choices for audio-based OSA screening. This observation aligns with the conclusions of a 2025 meta-analysis by Tan et al. [26].

Parallel to these algorithmic advancements, audio-based OSA screening methods can be broadly stratified into three levels of granularity. First, subject-level analysis extracts global features from the entire night’s recording to directly classify the patient’s risk status [18]. While this approach is efficient for rapid screening, it operates as a “black box,” failing to provide crucial clinical details such as the Apnea-Hypopnea Index (AHI) or the temporal distribution of respiratory events. Second, segment-level analysis divides audio into fixed-duration epochs (e.g., 30 s) to determine the presence of respiratory events within each segment [21,22,23]. Although this enables coarse-grained annotation of respiratory events and rough AHI estimation, it is prone to calculation errors driven by boundary effects, particularly where a single event is truncated across windows or a segment captures incomplete parts of multiple events. Third, snore-level analysis involves detecting isolated snore episodes and defining respiratory events through the post-processing of temporal patterns or intensity envelopes of snore clusters [24,27,28]. While this approach achieves finer granularity, its performance is fundamentally limited by the sensitivity of snore detection. Furthermore, relying exclusively on snoring sounds may lead to misclassification, as periods of quiet normal breathing between snores can be easily misinterpreted as apnea events.

In summary, while the aforementioned methods exhibit distinct limitations, they share a common critical deficiency: the inability to precisely delineate the onset and offset boundaries of respiratory events. To bridge this gap, this study proposes a fine-grained, lightweight detection framework utilizing a Convolutional Recurrent Neural Network (CRNN). By leveraging frame-level audio classification, we explore whether this approach can achieve real-time and high-precision identification of respiratory events relevant to OSA screening, thereby providing a solid foundation for accessible home-based OSA screening.

The remainder of this paper is organized as follows: Section 2 provides a comprehensive description of the study population and data acquisition protocols, followed by the data preprocessing pipeline. It details the methodological framework, including the rationale and design of the proposed dual-stream CRNN architecture, the hybrid loss function utilized for optimization, the post-processing strategy, and the specific evaluation metrics defined for frame-level, event-level, and patient-level assessment. Section 3 presents the comprehensive experimental results, covering frame-level classification, event-level temporal localization, and patient-level AHI estimation across both internal and external cohorts, followed by ablation studies, sensitivity analyses, and error analysis. Section 4 discusses the principal findings, compares performance against state-of-the-art methods, critically analyzes the challenges associated with hypopnea detection, and outlines limitations and future research directions. Finally, Section 5 summarizes the conclusions of the study.

## 2. Materials and Methods

### 2.1. Study Population and Data Acquisition

#### 2.1.1. The Sismanoglio Hospital Cohort (Internal Dataset)

The model was developed using the PSG-Audio dataset [29], which comprises 286 full-night recordings collected from adult patients (aged ≥18 years) at the Sismanoglio—Amalia Fleming General Hospital of Athens. Standard full-night PSG was conducted to establish the ground truth, recording a comprehensive set of physiological signals including electroencephalography (EEG), electrooculography (EOG), chin electromyography (EMG), electrocardiography (ECG), nasal airflow pressure, oronasal temperature, thoracic and abdominal respiratory effort, and pulse oximetry.

Simultaneously, audio signals were recorded in strict synchronization with the PSG data using a dual-microphone setup: (a) a contact microphone (CTH100; Clockaudio Ltd., Waterlooville, UK) placed on the patient’s trachea, and (b) an ultra-linear measurement condenser microphone (ECM8000; Behringer, Willich, Germany) placed approximately 1 m above the patient’s bed. Both sound signals were sampled at 48 kHz and stored as 24-bit uncompressed Waveform Audio Format (.wav) files. To simulate a non-contact home monitoring scenario, we exclusively utilized the audio channel recorded by the ambient microphone.

To ensure high reliability, the ground truth was established via a rigorous two-stage protocol: initial scoring by a certified technician followed by final validation by a senior sleep physician with over 30 years of experience to correct any missed or erroneous annotations. Patient participation was approved by the Local Ethics Committee of Sismanoglio Hospital, and data management strictly adhered to the European General Data Protection Regulation (GDPR).

It is important to note that this cohort consists of patients referred to the sleep center specifically for suspected sleep disorders, resulting in a class distribution heavily skewed towards severe pathology. Among the 286 subjects, the vast majority were diagnosed with severe OSA (*n* = 234), followed by moderate cases (*n* = 40). In contrast, the representation of mild OSA (*n* = 9) and healthy controls (*n* = 3) was extremely limited.

To address the class imbalance and ensure a representative evaluation during model development, the dataset was partitioned into three independent subsets using stratified random sampling based on OSA severity: a training set (70%, *n* = 200) for model optimization, a validation set (20%, *n* = 57) for hyperparameter tuning, and a test set (10%, *n* = 29) for final performance evaluation.

#### 2.1.2. The Beijing Tongren Hospital Cohort (External Dataset)

To evaluate the model’s performance and generalization capability in a real-world clinical scenario, we conducted an independent data collection at the Sleep Medicine Center of Beijing Tongren Hospital. We initially recruited 72 adult patients (aged ≥18 years) suspected of OSA for overnight monitoring.

Standard PSG was conducted using the Grael PSG system (Compumedics, Abbotsford, VIC, Australia), recording the identical set of physiological signals as described in the Sismanoglio Hospital Cohort. Simultaneously, audio data were captured using a Sony PCM-D10 non-contact digital recorder (Sony Corporation, Tokyo, Japan) positioned approximately 1 m above the participant’s head (Sample rate: 44.1 kHz, 16-bit resolution).

Following a rigorous data quality control process, 12 recordings were excluded due to synchronization failures or severe audio artifacts, resulting in a final cohort of 60 participants. Unlike the internal dataset, this independent cohort was specifically curated to achieve a relatively balanced distribution across AHI severity levels, facilitating a comprehensive diagnostic assessment. The final composition included: healthy controls (*n* = 9), mild OSA (*n* = 13), moderate OSA (*n* = 10), and severe OSA (*n* = 28). This balanced distribution allows for a rigorous assessment of the model’s screening effectiveness across the full spectrum of disease severity.

Sleep staging and respiratory event scoring were independently interpreted by two trained technicians according to standard clinical criteria. The study protocol was approved by the Institutional Review Board (IRB) of Beijing Tongren Hospital (Approval No. TREC2023-KY015).

### 2.2. Data Preprocessing

#### 2.2.1. Audio Resampling and Segmentation

To ensure consistency across datasets recorded at different sampling rates, all audio recordings were downsampled to 16 kHz. This sampling rate is sufficient to capture the relevant acoustic characteristics of snoring and breathing sounds while reducing computational complexity.

Subsequently, regarding temporal segmentation, we analyzed all respiratory events across the Sismanoglio Hospital Cohort and the Beijing Tongren Hospital Cohort. Our statistics revealed that the duration of respiratory events was 16.58 ± 6.68 s for hypopneas and 23.39 ± 10.10 s for apneas. According to the AASM Manual for the Scoring of Sleep and Associated Events (Version 3), a respiratory event is delineated from the nadir of the amplitude drop to the beginning of the recovery toward baseline. However, accurate interpretation fundamentally relies on comparing this amplitude reduction against a stable pre-event breathing baseline. Therefore, the detection window must encompass sufficient contextual information, typically spanning 2–3 breath cycles (approximately 10 s) before and after the event.

Based on this rationale, we selected a window size of 60 s with a 30 s stride (50% overlap). This design robustly captures the full event interpretation window, distinguishing our approach from the commonly used 5–30 s windows which risk truncating critical context.

The 50% overlap minimizes boundary truncation artifacts, and during inference, overlapping predictions are aggregated using temporal averaging. Ultimately, this segmentation process yielded a total of 118,417 clips for the training set, 31,248 clips for the validation set, and 17,074 clips for the test set from the Sismanoglio Hospital Cohort. In addition, the Beijing Tongren Hospital Cohort comprised 53,951 clips.

#### 2.2.2. Feature Extraction

The raw audio waveforms were transformed into Log Mel-spectrograms to serve as inputs for the CRNN model. The Short-Time Fourier Transform (STFT) was computed using the following parameters: a window size ($N_{FFT}$) of 1024, a hop length of 160 samples (corresponding to a fine temporal resolution of 10 ms), and 128 Mel bands ($N_{MELS}$). Consequently, each 60 s audio segment was converted into a feature map with dimensions of 128 × 6000, which was subsequently normalized to zero mean and unit variance.

Simultaneously, to explicitly capture signal intensity dynamics, we computed a frame-level energy profile. This was derived by calculating the mean intensity across all Mel frequency bands for each time step. The resulting 1D energy curve (length: 6000) was then normalized independently to serve as the input for the auxiliary branch of the network.

#### 2.2.3. Label Generation and Alignment

To enable fine-grained frame-level classification, we generated ground truth labels aligned with the temporal resolution of the spectrograms by mapping PSG annotations directly to the audio timeline. A three-class labeling scheme was adopted: for each time frame, if its timestamp fell within the annotated duration of a Hypopnea event, it was labeled as 1; if it coincided with an Apnea event (aggregating Obstructive, Central, and Mixed subtypes), it was labeled as 2; otherwise, it was labeled as 0. The consolidation of all apnea subtypes into a single class was implemented because Central and Mixed events constituted a negligible portion of the dataset, leading to extreme class imbalance that could otherwise hinder model convergence.

#### 2.2.4. Data Augmentation

To mitigate overfitting and enhance robustness to real-world acoustic variability, an online data augmentation strategy was applied exclusively during the training phase. Specifically, SpecAugment was employed on the Mel-spectrogram representation using frequency masking (maximum width F = 15) and time masking (maximum width T = 40), whereby contiguous regions along the frequency or temporal dimensions were randomly masked. This strategy discourages the model from over-reliance on localized time–frequency cues and promotes the learning of more robust contextual representations from partial observations.

In addition, to simulate variability in microphone sensitivity and patient positioning commonly encountered in non-contact recordings, random gain perturbation was applied. Both the input spectrogram and the auxiliary energy trajectory were multiplied by a gain factor sampled uniformly from U (0.8, 1.2), thereby preserving cross-modal consistency. Collectively, these augmentation techniques improve the model’s robustness to minor fluctuations in signal amplitude and recording conditions.

### 2.3. Dual-Stream CRNN Architecture

**Figure 1:**
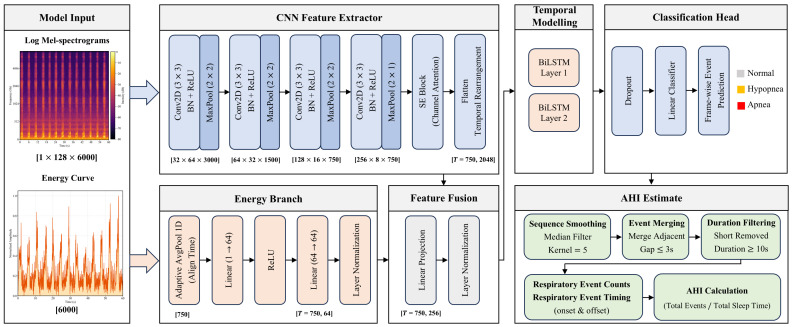
Schematic illustration of the proposed Dual-Stream CRNN architecture.

As illustrated in Figure 1, the proposed model adopts a Dual-Stream architecture that integrates spectral features with energy profiles to enhance the detection of respiratory events. The framework consists of four key components: a VGG-style Convolutional Neural Network (CNN) augmented with Squeeze-and-Excitation (SE) blocks, an auxiliary energy stream, a multi-modal fusion module, and a Bidirectional LSTM (BiLSTM) for temporal context encoding.

#### 2.3.1. Architecture Design Rationale

To ensure a balance between clinical precision and computational efficiency, our architectural design prioritizes the intrinsic acoustic characteristics of sleep breathing, specifically temporal continuity, event sparsity, and physiological intensity dynamics, over generic model complexity.

Regarding feature extraction, while models such as Whisper or Wav2Vec 2.0 offer powerful representations, they are fundamentally optimized for Automatic Speech Recognition with a focus on phoneme alignment and linguistic semantics. This focus creates a significant domain mismatch for physiological breathing acoustics. Such mismatch introduces not only functional redundancy but also excessive parameter overhead that contradicts our goal of edge deployment. Therefore, we adopted a VGG-style Convolutional Neural Network, which has been recognized for its efficacy in audio feature extraction [30]. Through preliminary experiments, we selected a 4-layer CNN depth. This depth strikes an optimal balance between extracting sufficiently abstract spectral features and avoiding the gradient degradation often observed in deeper networks. To further optimize features, we integrated Squeeze-and-Excitation blocks. By implicitly recalibrating feature weights via a channel-wise attention mechanism, this enables the network to selectively emphasize frequency bands relevant to airway obstruction while suppressing irrelevant background noise.

For temporal modeling, we selected a Bidirectional Long Short-Term Memory network over Transformer-based architectures or emerging State Space Models such as Mamba. Although Transformers offer global attention, their quadratic computational complexity allocates excessive resources to correlating distant time steps [31]. Since respiratory event detection primarily relies on identifying changes in the frequency or intensity patterns of breathing sounds and snores during the event and its adjacent periods, the global attention mechanism introduces redundancy. Furthermore, Transformers lack the inherent inductive bias required to model the strict temporal evolution of respiratory phases [32]. Similarly, while recent State Space Models offer linear complexity, their inherent causality limits the simultaneous integration of past and future contexts required for precise event boundary regression [33]. Additionally, models like Mamba often impose strict environmental requirements and entail complex deployment procedures, whereas their primary advantage lies in ultra-long sequence modeling. In contrast, BiLSTM provides native bi-directionality and stability suitable for the feature sequence length in this study. Specifically, the bidirectional structure allows for the integration of both past and future contexts, which is essential for delineating event boundaries [34]. Furthermore, the internal gating mechanisms enable the model to dynamically regulate information flow by preserving salient features associated with respiratory anomalies and discarding redundant data from steady breathing. This selective focus ensures precise event localization while maintaining linear computational complexity.

Finally, to address the acoustic ambiguity specific to hypopnea events, we introduced an auxiliary energy branch. Hypopnea is defined as a reduction in airflow of at least 30% rather than complete silence. Standard convolutional normalization often obscures these absolute intensity variations, rendering hypopneas spectrally indistinguishable from normal heavy breathing. By explicitly encoding the normalized energy profile as a secondary input, we force the model to attend to these fine-grained intensity dynamics. This strategy compensates for the limitations of relying solely on spectral features and enhances detection sensitivity for hypopneas, as detailed in the Ablation Study in Section 3.4.

#### 2.3.2. Spectrogram Encoding with SE-Attention

The primary branch processes Log Mel-spectrograms using a customized 4-layer VGG-style CNN. Each convolutional block consists of a 3 × 3 convolutional layer, Batch Normalization (BN), ReLU activation, and a Max-Pooling layer. To balance feature abstraction with temporal resolution, we designed a specific pooling strategy: The first three blocks utilize 2 × 2 Max-Pooling, progressively reducing the frequency dimension while resulting in an effective temporal resolution of 80 ms per frame. Crucially, the fourth block employs 2 × 1 Max-Pooling (pooling only along the frequency axis). This design preserves the temporal length of the feature sequence ($T_{seq}$) to align with the target label resolution. A Squeeze-and-Excitation (SE) block (reduction ratio r = 16) is embedded at the end of the CNN encoder to adaptively recalibrate channel-wise feature responses, emphasizing informative spectral patterns related to airway obstruction.

#### 2.3.3. Auxiliary Energy Encoding Branch

The proposed architecture incorporates the pre-extracted energy profile (described in Section 2.2.2) as a secondary input. This 1D signal is processed by a dedicated branch, where it is aligned to the CNN output timeline using adaptive average pooling and then projected into a 64-dimensional feature space via a Multi-Layer Perceptron (MLP) with Layer Normalization. By explicitly encoding these intensity dynamics, this branch provides cues to differentiate Hypopneas from normal snore clusters, compensating for the potential loss of fine-grained amplitude information during convolutional feature extraction.

#### 2.3.4. Multi-Modal Fusion and Temporal Modeling

The spectral features from the CNN (flattened to 2048 dimensions) and the energy features (64 dimensions) are concatenated and fused via a linear projection layer $d_{model}$ = 256 with Layer Normalization. The fused sequence is then fed into a 2-layer Bidirectional LSTM (BiLSTM). By processing the sequence in both forward and backward directions, the BiLSTM captures long-term temporal dependencies and contextual transitions between sleep breathing phases. Finally, a time-distributed fully connected layer outputs the frame-level classification logit.

### 2.4. Post-Processing Strategy

To translate the continuous frame-level probability maps generated by the model into discrete clinical events, we implemented a rigorous three-stage post-processing strategy. This process ensures that the detected events adhere to clinical duration constraints and mitigates the impact of signal instability.

Sequence Smoothing: The raw frame-level predictions often contain high-frequency fluctuations. To mitigate this “salt-and-pepper” noise, we applied a median filter (kernel size = 5) to the predicted label sequence, ensuring local continuity of the event detection.

Event Merging: Due to physiological variability or signal artifacts, a single long respiratory event might occasionally be fragmented into multiple segments separated by brief gaps. To address this, adjacent events of the same class separated by a gap of less than 3 s were merged into a single continuous event.

Duration Filtering: Clinical guidelines define an apnea or hypopnea event as a respiratory reduction lasting at least 10 s. Consequently, any candidate event with a duration shorter than 10 s after the merging step was discarded to strictly comply with standard scoring criteria.

To empirically verify the robustness of these parameter settings, a detailed sensitivity analysis was conducted, as presented in Section 3.5.2.

### 2.5. Hybrid Loss Function

To address the challenges of class imbalance (dominance of Normal breathing) and boundary ambiguity, we proposed a Hybrid Event Loss combining Focal Loss and Dice Loss:

$$L_{total}=L_{Focal}+\lambda \cdot L_{Dice}$$

Weighted Focal Loss: We modify the standard Cross-Entropy loss by introducing a focusing parameter γ = 2.0 and class-balancing weights α = 1.0, 4.0, 2.0 (for Normal, Hypopnea, Apnea, respectively). This forces the model to focus on hard-to-classify examples and minority classes.

Dice Loss: To ensure the structural continuity of predicted events, we employ a Dice Loss term that optimizes the overlap between predicted and ground-truth event masks. The weight factor λ was set to 0.5 to balance pixel-level classification accuracy with event-level overlap.

This hybrid formulation encourages both accurate frame-wise classification and coherent event-level segmentation.

### 2.6. Implementation Details

The proposed framework was implemented using PyTorch (version 2.9.1) and trained on a workstation equipped with an NVIDIA GeForce RTX 5070 Ti GPU. Reflecting a lightweight design, the model comprises a total of 1,734,147 trainable parameters. Optimization was performed using the AdamW algorithm with an initial learning rate of ${5\times 10}^{-4}$ and a weight decay of ${5\times 10}^{-2}$. To enhance training stability, we employed a ReduceLROnPlateau scheduler that halved the learning rate upon a plateau in validation F1-score lasting two consecutive epochs. Gradient clipping with a maximum norm of 1.0 was utilized to prevent exploding gradients in the recurrent layers, while Automatic Mixed Precision (AMP) was adopted to accelerate computation. Consequently, the model demonstrated robust computational efficiency, achieving an inference speed of approximately 9.96 iterations per second during the validation phase. Additionally, Dropout (*p* = 0.2) was applied within the BiLSTM layers and preceding the final classification head to prevent overfitting. Early stopping was applied based on validation macro-F1 score, and the model achieving the best validation performance was selected for final evaluation. Detailed hyperparameters are provided in Supplementary Table S1.

### 2.7. Evaluation Metrics

#### 2.7.1. Frame-Level Classification Performance

For the frame-wise classification task (Normal vs. Hypopnea vs. Apnea), we utilized standard metrics including Precision (Pre), Recall (Rec), and F1-score (F1). Given the class imbalance inherent in sleep data, we reported the macro-averaged F1-score (Macro-F1) to treat all classes equally, ensuring that the minority classes (Hypopnea and Apnea) significantly contribute to the overall performance evaluation. These metrics are defined as follows:

$$Pre_{c}=\frac{TP_{c}}{TP_{c}+FP_{c}}$$

$$Rec_{c}=\frac{TP_{c}}{TP_{c}+FN_{c}}$$

$$F1_{c}=\frac{2\cdot Pre_{c}\cdot Rec_{c}}{Pre_{c}+Rec_{c}}$$

$$Macro\text{-}F1=\frac{1}{C}\sum_{c=1}^{C}F1_{c}$$

where $TP_{c}$, $FP_{c}$, and $FN_{c}$ represent the true positives, false positives, and false negatives for class c, and C = 3 is the number of classes.

#### 2.7.2. Event-Level Temporal Localization Metrics

To rigorously validate the “fine-grained” capability of our model and quantify the precision of event boundaries, we introduced segmentation-based metrics beyond standard classification scores:

Intersection over Union (IoU): This metric measures the overlap between the predicted event segments and the ground truth annotations, and is calculated as the ratio of the intersection area to the union area of the matched event pairs. To ensure rigorous evaluation, a greedy matching strategy was employed, whereby a predicted event was considered a valid match for a ground truth event only if it maximized the overlap and exceeded a minimum IoU threshold of 0.1.

$$\mathrm{IoU}=\frac{\mathrm{Area}\left(\mathrm{Prediction}\cap \mathrm{Ground}\;\mathrm{Truth}\right)}{\mathrm{Area}\left(\mathrm{Prediction}\cup \mathrm{Ground}\;\mathrm{Truth}\right)}$$

Boundary Alignment Error: To evaluate the temporal shift, we calculated the Mean Absolute Error (MAE) for the event onset (start time) and offset (end time) in seconds. This metric quantifies the average temporal deviation of the detected boundaries relative to the gold standard for all correctly matched event pairs.

#### 2.7.3. Patient-Level AHI Estimation and OSA Screening

Based on the valid respiratory events identified by the post-processing strategy, the Audio-AHI for each subject was calculated as the total number of detected apnea and hypopnea events divided by the total sleep duration (in hours). To comprehensively evaluate the clinical utility of the proposed framework, we employed a multi-dimensional assessment strategy covering both continuous estimation and binary screening.

AHI Estimation Agreement: We assessed the concordance between the predicted Audio-AHI and the ground truth PSG-AHI using the Pearson Correlation Coefficient (r), Mean Absolute Error (MAE), and Bland–Altman analysis to quantify the limit of agreement (LoA).

OSA Screening Performance: We evaluated the model’s capability to screen for OSA at standard clinical severity thresholds: Mild (AHI ≥ 5 events/h), Moderate (AHI ≥ 15 events/h), and Severe (AHI ≥ 30 events/h). For each threshold, standard screening metrics were reported, including Sensitivity, Specificity, Accuracy, Positive Predictive Value (PPV), Negative Predictive Value (NPV), Cohen’s Kappa coefficient (k), and the Area Under the Receiver Operating Characteristic Curve (AUC). To quantify the statistical reliability of these results, 95% confidence intervals (CIs) for all reported metrics (including correlation, MAE, and screening statistics) were calculated using the non-parametric bootstrap method with 1000 iterations. However, for classification metrics yielding 100% performance, Clopper-Pearson exact confidence intervals were reported to provide a conservative estimate of uncertainty given the sample size.

## 3. Results

### 3.1. Frame-Level Classification Performance (Internal Test Set)

The frame-level classification performance of the proposed Dual-Stream CRNN on the internal test set (Sismanoglio Cohort) is summarized in Table 1. The model achieved a macro-averaged F1-score of 0.64 and an overall accuracy of 0.81.

For Apnea events, the model exhibited robust performance, achieving a precision of 0.74 and a recall of 0.71. This indicates the model’s effectiveness in characterizing periods of complete airflow cessation, which are acoustically distinct. The Normal breathing segments were identified with high reliability, yielding an F1-score of 0.89, ensuring a low false-positive rate for healthy breathing intervals.

As anticipated, Hypopnea detection proved more challenging due to the high acoustic similarity between hypopnea-associated snoring and normal snoring. Consequently, the model achieved an F1-score of 0.31 for this class. However, notably, the recall for Hypopnea was 0.41. This sensitivity is a significant achievement for granular frame-level detection, as it ensures that a sufficient number of subtle respiratory reduction events are captured, providing valuable cues for the subsequent post-processing and patient-level AHI estimation.

**Figure 2:**
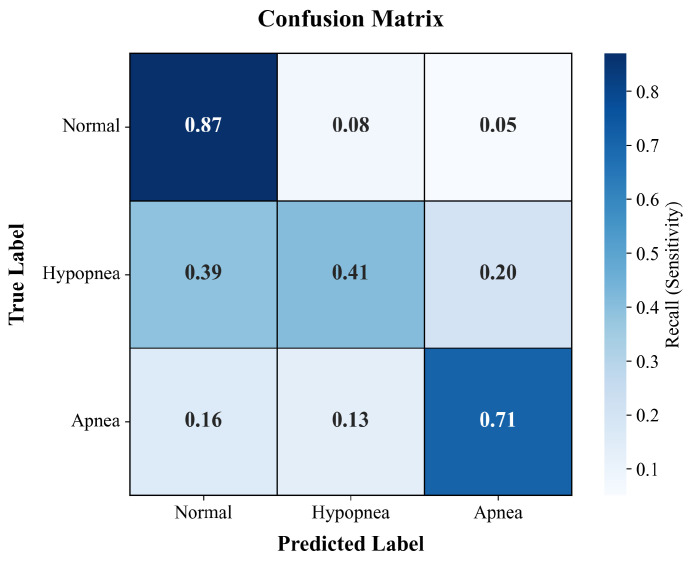
Confusion matrix of frame-level classification on the internal test set.

The confusion matrix, illustrated in Figure 2, further visualizes the distribution of predictions. It can be observed that the primary source of error stems from the misclassification between Hypopnea and Normal classes.

### 3.2. Event-Level Temporal Localization and Qualitative Analysis (Internal Test Set)

Beyond frame-level statistics, we quantitatively evaluated the model’s capability to localize discrete events. After delineating individual event boundaries from the frame-level outputs via post-processing, we calculated the Intersection over Union (IoU) and boundary deviations against manual annotations. On the internal test set, the detected events achieved an Average IoU of 0.82, indicating a high degree of overlap. Furthermore, the temporal alignment was precise, with a Mean Absolute Error (MAE) of 1.49 s for onset and 1.12 s for offset. Given the typical 10–40 s duration of respiratory events, these deviations confirm the model’s accuracy in capturing the initiation and termination of airflow reduction.

**Figure 3:**
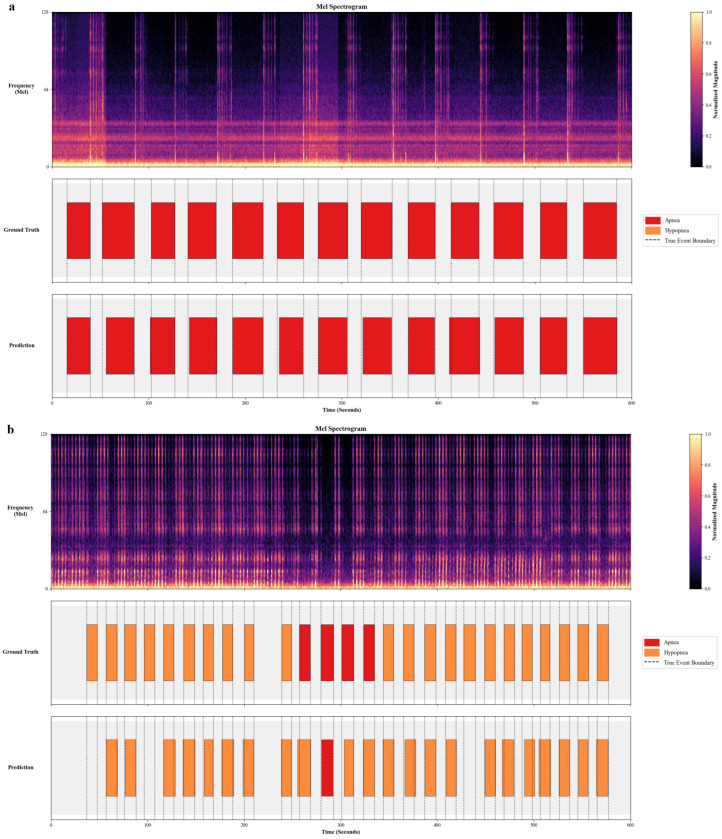
Visual alignment between automated detection and manual scoring for respiratory events. (**a**) Patient segment dominated by apnea events; (**b**) patient segment dominated by hypopnea events.

To visually corroborate these metrics, Figure 3 compares model predictions with manual annotations for two internal test subjects.

Figure 3a displays a segment dominated by Apnea events. The model achieved precise temporal alignment, accurately delineating the contiguous apnea episodes (red segments) which correspond to silence intervals.

Figure 3b illustrates a Hypopnea-dominant scenario. The model demonstrated strong capability in localizing the majority of respiratory events. While the overall event structure was correctly captured, minor discrepancies were observed, including the omission of some subtle, low-intensity hypopneas and occasional misclassification between event types. This reflects the inherent acoustic challenge in distinguishing the fine-grained severity between partial and complete airway obstruction.

### 3.3. Patient-Level AHI Estimation and OSA Screening (Internal Test Set)

**Figure 4:**
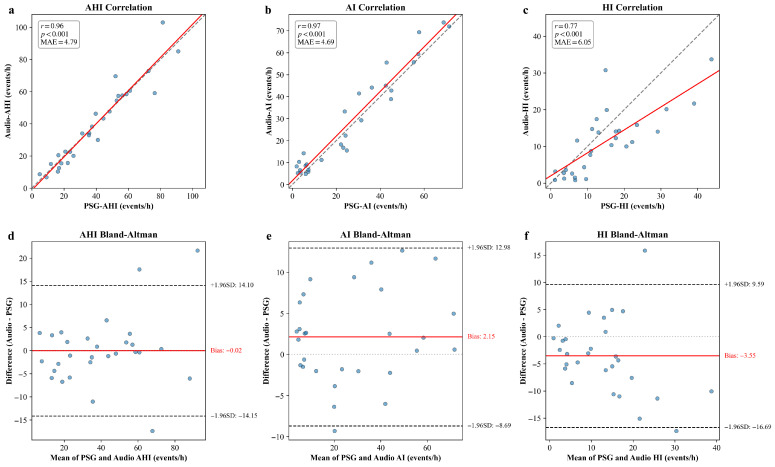
Comparison of audio-derived respiratory indices with ground truth PSG measurements using scatter plots and Bland–Altman analysis in internal test set. (**a**) Scatter plot for AHI; (**b**) scatter plot for AI; (**c**) scatter plot for HI; (**d**) Bland–Altman plot for AHI; (**e**) Bland–Altman plot for AI; (**f**) Bland–Altman plot for HI.

Building upon the discrete respiratory events identified in the previous step, we further calculated the patient-level clinical metrics. specifically, we derived the Apnea-Hypopnea Index (AHI), Apnea Index (AI), and Hypopnea Index (HI) for each subject in the test set by normalizing the event counts over the total sleep duration. Figure 4 illustrates the agreement between the audio-derived indices and the ground truth PSG indices.

The scatter plots (Figure 4a–c) reveal strong positive linear correlations across all three metrics. The model achieved a Pearson correlation coefficient (r) of 0.96 (95% CI: 0.93–0.98) for AHI, with a Mean Absolute Error (MAE) of 4.79 events/h (95% CI: 3.66–5.95). Notably, the AI demonstrated strong agreement (r = 0.97 [95% CI: 0.94–0.99], MAE = 4.69 events/h [95% CI: 3.53–5.86]), reflecting the distinct acoustic characteristics of apnea events. The HI, while more challenging due to the acoustic subtlety of the events, still maintained a substantial correlation (r = 0.77 [95% CI: 0.60–0.88], MAE = 6.05 events/h [95% CI: 4.72–7.31]).

To further assess the agreement, Bland–Altman analysis was conducted (Figure 4d–f). For AHI, the mean bias was −0.02 events/h, indicating a negligible bias between the model predictions and ground truth. Furthermore, the majority of the data points fell within the 95% Limits of Agreement (LoA: −14.15 to 14.10 events/h), suggesting that the automated estimations are clinically consistent with standard PSG scoring. Similarly, the bias for specific event types remained within a reasonable range, with AI showing a bias of 2.15 events/h and HI showing a bias of −3.55 events/h, confirming that the model does not exhibit significant systematic errors in classifying specific event types.

Regarding screening on this internal test set, the model demonstrated excellent capability in identifying Severe OSA (AHI ≥ 30). For this critical threshold, the model achieved an accuracy of 96.55% (95% CI: 89.66–100.00%), a sensitivity of 94.44% (95% CI: 83.33–100.00%), a specificity of 100.00% (95% CI: 71.5–100.0%), and an AUC of 0.99 (95% CI: 0.96–1.00). This indicates that despite the dataset’s bias, the model can reliably distinguish severe patients requiring immediate intervention from non-severe cases. However, it is important to note that the internal dataset exhibits a scarcity of healthy controls and mild OSA patients, which is characteristic of clinical referral cohorts. Consequently, calculating screening specificity at lower thresholds (AHI ≥ 5 and 15) on this test set would yield statistically unreliable results due to the insufficient sample size of true negative cases. Therefore, to provide a rigorous and clinically meaningful evaluation of the model’s full screening capability across all severity levels, we detail the comprehensive screening performance in the External Cohort analysis (Section 3.6).

### 3.4. Ablation Study: Impact of Dual-Stream and Temporal Modeling

To verify the contribution of the key components in our proposed framework, we conducted an ablation study on the internal test set. We compared the performance of the complete model against two variants: (1) *w*/*o* BiLSTM: A baseline CNN model utilizing only spectrogram features without temporal modeling; and (2) *w*/*o* Energy Stream: The CRNN model excluding the auxiliary energy input.

Table 2 summarizes the quantitative results. The baseline CNN-only model yielded a significantly lower performance, with a Macro Recall of only 0.37 and a Macro F1 of 0.36. The integration of the BiLSTM module (*w*/*o* Energy variant) resulted in a significant performance improvement, increasing the Recall to 0.64. This confirms that capturing long-term temporal dependencies is essential for characterizing respiratory events, which are inherently defined by their duration and evolution over time.

Furthermore, the incorporation of the auxiliary Energy stream provided a consistent improvement across all metrics. As shown in Table 2, the proposed Dual-Stream framework achieved the highest Accuracy (0.81) and Macro F1 (0.64). Compared to the single-stream counterpart, the addition of energy descriptors improved the Recall by 0.02. This increment suggests that explicitly modeling signal intensity aids the network in distinguishing subtle airflow reductions (typical of hypopnea) that might be ambiguous in the spectral domain alone, thereby enhancing the model’s overall sensitivity.

### 3.5. Hyperparameter Analysis and Robustness Verification

#### 3.5.1. Impact of Input Window Duration on Performance and Efficiency

To empirically validate the optimal input duration derived from our statistical analysis (Section 2.2.1), we evaluated the model’s performance across a range of window sizes: 5 s, 30 s, 60 s, 90 s, and 120 s. Supplementary Table S2 details the trade-off between diagnostic accuracy and edge deployment metrics.

Results indicate that the 5 s window yielded the lowest performance, primarily because it fails to capture the minimum duration (≥10 s) required to clinically define a respiratory event. Increasing the window to 30 s resulted in a marked improvement, as this duration covers the majority of event occurrences; however, it still lagged behind the 60 s configuration, suggesting that 30 s provides insufficient surrounding context for precise characterization. Performance saturated at 60 s, with further extensions to 90 s and 120 s yielding no significant gains while substantially prolonging inference time. Regarding deployment feasibility, the 60 s window retains a constant parameter count (1.73 M) and a computational density of 0.40 GFLOPs/s. With a CPU latency of 84.81 ms, the resulting processor duty cycle is approximately 0.14%. These metrics indicate that the model remains suitable for continuous edge monitoring despite the increased input size.

#### 3.5.2. Sensitivity Analysis of Post-Processing Parameters

To evaluate the robustness of post-processing parameters, we performed a sensitivity analysis on median filter size, gap-merging threshold, and minimum duration (Supplementary Table S3). Variations in median filter size (3–10) produced negligible deviations in metrics. In contrast, increasing the gap-merging threshold to 8 s reduced the IoU to 0.802 and increased the AHI MAE to 5.29; this suggests that excessive gap tolerance merges distinct respiratory events, resulting in boundary errors and AHI underestimation. Regarding minimum duration, an 8 s threshold yielded the lowest statistical error (MAE = 4.65). This improvement occurs because the model may predict the duration of short respiratory events slightly below their ground truth; an 8 s cutoff effectively recovers these valid detections, thereby enhancing sensitivity. However, strictly adhering to the AASM Manual for the Scoring of Sleep and Associated Events (Version 3), which mandates a duration of ≥10 s for respiratory events, we selected the 10 s threshold. This decision prioritizes clinical compliance over marginal statistical gains, ensuring the system generates diagnostically valid indices despite a slight increase in MAE (4.79).

### 3.6. External Validation: Generalization and Screening Robustness

To validate the robustness of the proposed model against domain shifts caused by distinct recording environments and microphone equipment, we conducted a comprehensive evaluation on the independent Beijing Tongren Hospital cohort.

Event-level Temporal Localization: Before assessing clinical indices, we verified the model’s fundamental capability to detect and localize respiratory events in unseen acoustic domains. Despite differences in background noise profiles and device frequency responses, the model maintained precise temporal alignment with manual annotations. Quantitatively, it achieved an Average IoU of 0.78 on this external set. Furthermore, the boundary detection remained accurate, yielding a Mean Absolute Error (MAE) of 1.89 s for onset and 1.63 s for offset. These results confirm that the learned features effectively capture the intrinsic acoustic signatures of airflow reduction rather than overfitting to source-specific artifacts.

**Figure 5:**
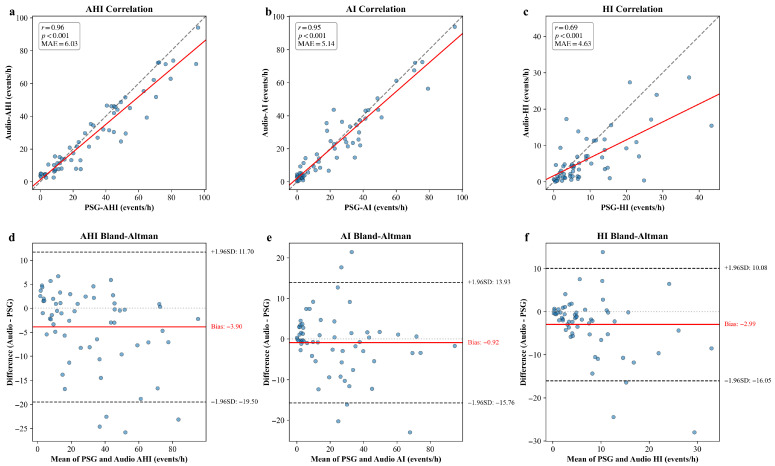
Comparison of audio-derived respiratory indices with ground truth PSG measurements using scatter plots and Bland–Altman analysis in external set. (**a**) Scatter plot for AHI; (**b**) scatter plot for AI; (**c**) scatter plot for HI; (**d**) Bland–Altman plot for AHI; (**e**) Bland–Altman plot for AI; (**f**) Bland–Altman plot for HI. Red solid lines for regression or bias, dashed lines for identity or limits of agreement.

Patient-level AHI Estimation and OSA Screening: Building on the robust event detection, we assessed the consistency of continuous estimation metrics. Figure 5 illustrates the agreement between the model-derived indices and the reference PSG metrics.

The scatter plots (Figure 5a–c) reveal strong positive linear correlations across all three metrics. The model demonstrated strong linear correlations for AHI (r = 0.96 [95% CI: 0.93–0.97]) with a MAE of 6.03 events/h (95% CI: 4.61–7.78). Furthermore, the model exhibited robust generalization in quantifying specific event types. The AI achieved a correlation of r = 0.95 (95% CI: 0.90–0.97) with a MAE of 5.14 events/h (95% CI: 3.83–6.55), and the HI yielded a correlation of r = 0.69 (95% CI: 0.44–0.85) with a MAE of 4.63 events/h (95% CI: 3.31–6.10). The Bland–Altman analysis (Figure 5d–f) further confirms that the vast majority of predictions across all three metrics fall within the 95% limits of agreement, validating the model’s reliability for quantitative assessment in diverse clinical settings.

Screening Capability: Finally, we evaluated the binary screening performance across standard clinical thresholds (Table 3). Crucially, the relatively balanced distribution of this external cohort allowed for an unbiased assessment of specificity, particularly for mild disease.

Mild OSA (AHI ≥ 5): The model achieved an accuracy of 96.67% (95% CI: 91.67–100.00%) and a specificity of 88.89% (95% CI: 62.50–100.00%). Additionally, the model demonstrated a sensitivity of 98.04% (95% CI: 93.88–100.00%) and an AUC of 0.97 (95% CI: 0.90–1.00). This result validates the model’s capability to effectively differentiate symptomatic patients from healthy individuals, successfully addressing the limitation of the internal dataset regarding negative sample evaluation.

Moderate (AHI ≥ 15) and Severe (AHI ≥ 30) OSA: The model exhibited sustained high performance, with AUCs of 0.97 (95% CI: 0.93–1.00) and 1.00 (95% CI: 0.99–1.00), respectively. Notably, at the AHI ≥ 30 threshold, the high sensitivity (89.29% [95% CI: 76.92–100.00%]) and specificity (100.00% [95% CI: 88.43–100.00%]) confirm the model’s reliability in prioritizing severe cases for immediate intervention, consistent with the findings in the internal validation.

### 3.7. Error Analysis of Hypopnea Detection

While the proposed framework achieved robust performance in assessing the overall respiratory burden, the evaluation of hypopnea remains less satisfactory. To further investigate the challenges in hypopnea detection, we conducted a quantitative error analysis on the external test set. Preliminary statistics revealed a notable recall deficit: the model predicted only 2226 hypopnea events against a ground truth of 3300. To pinpoint the sources of this discrepancy, events were matched and categorized following the IoU-based greedy strategy defined in the Materials and Methods Section.

Hypopnea misclassified as Normal accounted for the largest proportion of errors (39%), representing the primary source of under-detection. Hypopnea misclassified as Apnea constituted 27% of the discrepancies. Conversely, false positive errors, specifically Normal misclassified as Hypopnea and Apnea misclassified as Hypopnea, accounted for 26% and 8%, respectively. The underlying mechanisms of these misclassifications, including acoustic ambiguity and labeling uncertainty, are further discussed in the Discussion Section.

## 4. Discussion

### 4.1. Principal Findings

The primary objective of this study was to develop a fine-grained and lightweight audio-based framework for obstructive sleep apnea diagnosis that is capable of providing clinically interpretable outputs. Specifically, the proposed framework enables relatively accurate patient-level AHI estimation, as well as fine-grained characterization of respiratory events, including the onset and offset times, durations, and temporal distribution. Evaluated across multiple levels of granularity and validated on two independent cohorts, the proposed Dual-Stream CRNN demonstrated robust and consistent performance for audio-based OSA screening and severity assessment.

On the internal PSG-Audio cohort, the model exhibited strong fine-grained detection capability. At the frame level, it achieved a macro-averaged F1-score of 0.64, with a recall of 0.41 for hypopnea events, which are widely recognized as the most acoustically ambiguous respiratory disturbances. This frame-wise sensitivity enabled accurate reconstruction of individual respiratory events, resulting in accurate temporal localization with a high average Intersection over Union (IoU = 0.82) and minimal onset and offset deviations. Importantly, this event-level precision translated into reliable patient-level quantification, as reflected by a strong correlation between the audio-derived AHI and PSG-based AHI (r = 0.96).

On the independent Beijing Tongren Hospital cohort, the model further demonstrated robust generalization in the presence of domain shifts arising from different recording devices and acoustic environments. Despite these variations, the framework maintained stable screening performance across OSA severity levels. In particular, for mild OSA, the model achieved a specificity of 88.89%, supporting its role as a safe screening tool with a low false-positive rate. At the severe OSA threshold, it achieved perfect specificity and high sensitivity, indicating reliable identification and prioritization of patients at highest clinical risk.

Collectively, these results indicate that the proposed framework not only supports accurate OSA screening but also enables fine-grained temporal analysis and clinically meaningful severity estimation using non-contact sleep audio.

### 4.2. Challenges in Hypopnea Detection and Clinical Implications

While the proposed framework achieved robust performance in assessing the overall respiratory burden, hypopnea detection exhibited relatively lower performance. We hypothesize that this limitation may arise from a combination of data imbalance, acoustic ambiguity, and constraints imposed by current clinical labeling protocols.

First, the model faces a hierarchical class imbalance. In most patients with OSA, the frequency of respiratory events is substantially lower than that of non-respiratory segments. Moreover, the dataset exhibits an Apnea-to-Hypopnea ratio of approximately 4:1. Although we employed a Weighted Focal Loss to penalize hard examples and up-weight the minority hypopnea class, this scarcity necessitates a delicate trade-off: aggressive re-weighting to improve recall risks amplifying false positives, which is counterproductive given the limited sample size.

**Figure 6:**
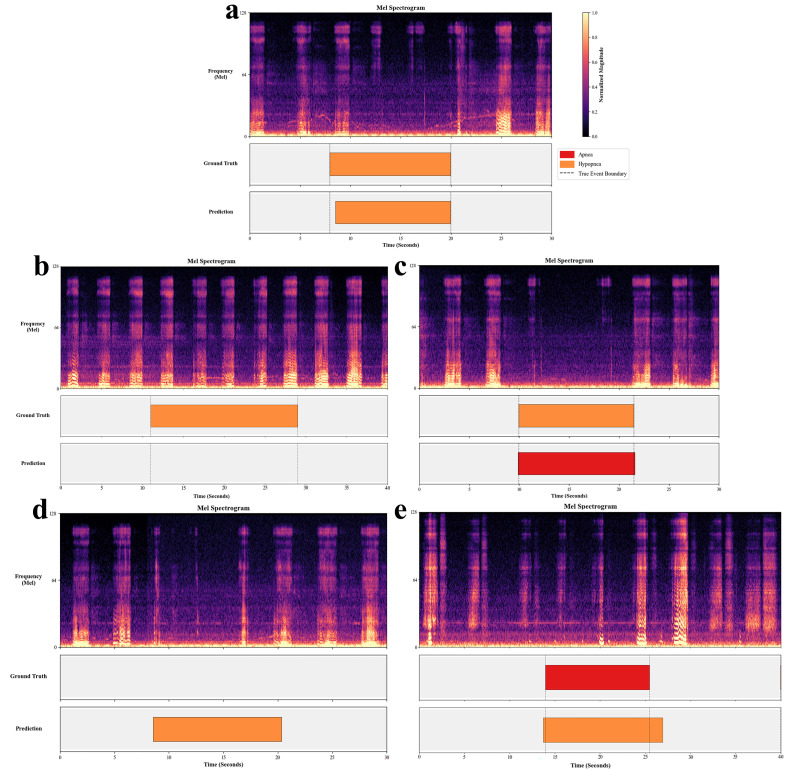
Representative spectrograms illustrating correct hypopnea detection and four characteristic misclassification patterns. (**a**) Correctly Classified Hypopnea (**b**) Hypopnea Misclassified as Normal (**c**) Hypopnea Misclassified as Apnea (**d**) Normal Misclassified as Hypopnea (**e**) Apnea Misclassified as Hypopnea.

Second, beyond data distribution, a major challenge lies in the acoustic definition of hypopnea itself, which directly shapes the error patterns observed in our results. Distinguishing hypopnea (≥30% flow reduction) from normal segments is far subtler than detecting the near-silence of apnea. A correctly classified hypopnea, as shown in Figure 6a, typically manifests as continuous snoring with a perceptible intensity dip. However, the ≥30% flow reduction is an arbitrary human-defined threshold rather than a binary acoustic turning point. Consequently, the absence of significant frequency/energy shifts accounts for the highest proportion of errors (Hypopnea misclassified as Normal, 39%). As illustrated in Figure 6b, these missed events (mild hypopneas) are acoustically indistinguishable from regular heavy breathing, lacking the clear boundaries required for detection. Conversely, as the degree of obstruction intensifies, snore loudness drops significantly, mimicking the silence of apnea [35]. This resemblance leads the model to misclassify severe hypopneas as apneas (accounting for 27% of errors), as shown in Figure 6c.

Third, a critical limitation of audio-only screening is the inability to access the multimodal data required for strict AASM compliance. While audio models can detect acoustic variations stemming from airflow limitation, they cannot verify the physiological consequences (≥3% oxygen desaturation or arousal) required for clinical confirmation of hypopnea events. This inherent lack of physiological context leads to false positives, specifically Normal misclassified as Hypopnea (26%), as illustrated in Figure 6d, where a segment of low-intensity snoring is erroneously identified as a hypopnea event. Similarly, when Apnea is misclassified as Hypopnea (8%), shown in Figure 6e, residual rhythmic low-intensity signals are visible during the apnea event, likely reflecting respiratory effort. These ambiguous features mislead the model and result in incorrect classification.

Finally, the inherent subjectivity of polysomnography (PSG) scoring contributes to inter-technician variability, which acts as a confounding factor in model evaluation. As a labor-intensive process, PSG scoring relies on the manual interpretation of physiological signals; consequently, subtle discrepancies in visually estimating whether airflow reduction meets the ≥30% threshold for hypopnea or the ≥90% threshold for apnea, or in identifying micro-arousals, can lead to inconsistencies in hypopnea annotation. Although formal inter-scorer agreement statistics were not quantified for the cohorts in this study, data reliability was strictly safeguarded through a two-tier validation process: all recordings underwent initial scoring by a certified technician, followed by a final, comprehensive review by a senior sleep physician with extensive clinical expertise. Furthermore, data collection was conducted at a specialized sleep medicine center, where all recordings were scored in strict adherence to the AASM Manual for the Scoring of Sleep and Associated Events, ensuring that the ground-truth labels meet high clinical standards. In future studies, to further mitigate labeling uncertainty, stricter multi-rater consensus strategies should be implemented, and inter-technician consistency should be formally evaluated to refine the granularity of event classification.

Despite these challenges, the overall assessment of AHI remains robust (r = 0.96), which may be attributed to the lower prevalence of hypopnea in our dataset and the error cancellation mechanism. Specifically, our error analysis indicated that among the identified misclassifications, a substantial proportion (27%) involved hypopneas being labeled as apneas due to acoustic similarities in severe obstruction. Since the AHI is an aggregate index that sums all respiratory events regardless of subtype, these specific errors do not alter the final event count, effectively preserving the correlation with PSG. This phenomenon aligns with findings from previous audio-based OSA studies [21,22,23], which similarly observed strong AHI alignment despite reduced subtype precision.

However, a critical distinction must be drawn between “misclassification” and “missed detection.” While the error cancellation mechanism protects the AHI against severity label permutations, it offers no defense against the complete under-detection of mild hypopneas (which accounted for 38% of the total errors). The high overall correlation observed in this study likely reflects the specific demographic of our cohort, characterized by moderate-to-severe male patients with an “Apnea-dominant” phenotype, where the sheer volume of correctly identified apneas masks the impact of missed mild hypopneas. Consequently, in cohorts where hypopnea is the dominant event type (e.g., female patients or milder OSA cases), this masking effect would diminish, exposing the risk of AHI underestimation and potential severity downgrading. Future research should aim to curate datasets balanced not only in disease severity but also in respiratory event phenotypes to ensure generalized clinical utility across diverse patient populations.

### 4.3. Comparison with Previous Studies Based on the PSG-Audio Dataset

To rigorously benchmark the proposed framework, we compared our results with existing studies that employed ambient microphone recordings from the same PSG-Audio dataset for OSA detection. It should be noted that these studies mainly reported internal evaluations, while external validation was not explored, leaving the generalization performance in real-world clinical settings less examined.

Salvatore et al. [36] utilized a pretrained Google VGGish model for feature extraction, combined with a Bi-LSTM classifier to determine whether 5 s audio segments contained respiratory events. By aggregating predictions via a 1 s sliding window, they provided a coarse estimation of the full-night respiratory events. However, their best model achieved an AHI correlation of only 0.76, which is significantly lower than the 0.96 reported in this study, and it lacked the capability to differentiate between event subtypes. A primary limitation lies in the temporal context: their 5 s input duration, constrained by the standard Google VGGish architecture, is insufficient to capture the complete evolution of respiratory events. In contrast, our framework employs a 60 s window with a 30 s stride. This design strikes an optimal balance, capturing full event patterns without the excessive computational overhead associated with processing extremely long sequences.

Korompili et al. [17] adopted a multi-stage, rule-based signal processing pipeline. Their approach involved dividing audio into 0.1 s frames, estimating breathing probabilities via Voice Activity Detection (VAD) and Hidden Markov Model (HMM), and identifying events using heuristic rules based on fixed energy thresholds, duration constraints, and minimum intervals. Due to dataset imbalance, they only reported binary classification results for moderate (AHI ≥ 15) and severe (AHI ≥ 30) thresholds. The performance was suboptimal, yielding sensitivities of only 55.4% and 65.6%, with notably low Positive Predictive Values (PPV) of 21.3% and 50%, respectively. While this method also operates at the frame-level, its performance is severely constrained by strong prior assumptions. Specifically, fixed thresholds (e.g., ≥50% RMS energy drop) fail to capture the acoustic heterogeneity of OSA, particularly for hypopneas. Furthermore, energy-driven rules are inherently susceptible to background noise, leading to high false-positive rates and limited generalization. Conversely, our Dual-Stream CRNN employs a data-driven approach, jointly modeling spectral features and energy dynamics. This allows the model to learn flexible, non-linear decision boundaries, resulting in superior screening performance across all thresholds.

Most recently, Kim et al. [25] proposed ApneaWhisper, innovatively reframing OSA detection as a token-based semantic segmentation task. Leveraging a fully fine-tuned pre-trained Whisper speech recognition backbone, this model encodes 20 s spectrogram clips and utilizes a Transformer-based autoregressive decoder to generate discrete tokens (e.g., “START_OSA”, “END_HYPOPNEA”). However, the reported frame-level performance suggests certain limitations regarding clinical applicability.

First, the model demonstrated limited specificity in distinguishing normal breathing. The Recall for Normal segments was 58.0%, with a notable proportion of normal segments misclassified as respiratory events. Specifically, 25.4% as Hypopnea and 14.8% as OSA. From a clinical perspective, this tendency toward false positives in healthy segments could theoretically contribute to AHI overestimation, particularly in individuals with low disease burden. In contrast, our proposed framework achieved a Normal Recall of 87%, reducing the rate of false alarms.

Second, the sensitivity for subtle events in the token-based baseline model remained constrained, as evidenced by a Hypopnea Recall of 29.5%. This indicates that a substantial portion of hypopnea frames were not detected, suggesting a risk of AHI underestimation in patients with hypopnea-predominant phenotypes. In comparison, our model achieved a Hypopnea Recall of 41%. Although identifying mild flow reductions remains challenging due to acoustic ambiguity, our approach retrieves a larger proportion of valid events compared to the token-based baseline.

A 20 s non-overlapping window is insufficient for capturing the full temporal evolution of respiratory events, which frequently exceed this duration. However, this design choice was an inherent trade-off to accommodate the substantial parameter size of the Whisper model. Since the Transformer’s self-attention mechanism scales quadratically with sequence length ($O\left(L^{2}\right)$), processing longer sequences at the model’s native 10 ms resolution would result in prohibitive computational costs. Furthermore, a domain mismatch exists: Whisper is optimized for speech recognition, introducing functional redundancy when applied to the semi-periodic, physiological patterns of breathing. In contrast, our Dual-Stream CRNN architecture employs a customized CNN to extract high-resolution spectral features, effectively capturing the fine-grained acoustic signatures of breathing and snoring. Synergistically combined with an auxiliary energy stream that explicitly encodes signal intensity dynamics, this lightweight feature representation allows the BiLSTM to efficiently leverage extended 60 s segments with overlapping strides. This approach not only captures the complete temporal context of respiratory cycles but also utilizes gating mechanisms (i.e., forget and update gates) to selectively focus on relevant state transitions while filtering out inter-breath noise, ultimately achieving superior performance with significantly lower computational overhead.

Furthermore, to objectively justify the “lightweight” designation, we benchmarked the inference latency of the aforementioned CRNN model by Serrano et al. and the Transformer-based ApneaWhisper on the same GPU used in our study. It should be noted that the baseline metrics presented here are estimates derived from architectures reproduced according to the specifications detailed in their respective studies.

For the CRNN-based model by Serrano et al. (5.98 M parameters), the measured inference latency was 172.92 ms/min. This metric is primarily driven by the sliding-window mechanism with a 1 s stride, which necessitates 60 separate forward passes per minute, resulting in cumulative computational overhead. Regarding the Transformer-based ApneaWhisper (40.17 M parameters), the latency was recorded at 146.57 ms/min. While this architecture benefits from hardware parallelism, its efficiency remains inherently constrained by the iterative nature of autoregressive decoding.

In comparison, our Dual-Stream CRNN (1.73 M parameters) achieves a total latency of only 11.60 ms/min. By processing the full 60 s context in a single forward pass, our architecture avoids both the repetitive overhead of sliding windows and the sequential bottleneck of autoregressive generation, making it highly suitable for resource-constrained edge deployment.

### 4.4. Comparison with State-of-the-Art Non-Contact OSA Screening Methods

To benchmark our model against current state-of-the-art methods, we compiled data from representative non-contact audio-based studies published within the past five years. We focused exclusively on literature that explicitly documented sensitivity and specificity across varying AHI thresholds, allowing for a direct performance assessment against our proposed framework. Table 4 summarizes the sensitivity and specificity across three clinical thresholds (AHI ≥ 5, 15, and 30 events/h). It should be noted that these comparisons are intended for reference only, as the included studies differ substantially in dataset characteristics, including sample size, distribution of AHI severity, ethnic composition, demographic profiles, and related factors. Such variations may influence reported performance and limit the direct comparability of results across studies. Importantly, our study included external validation across independent cohorts, which may enhance the robustness and generalizability of the reported results compared with studies evaluated on single center or homogeneous datasets.

For mild OSA screening, high sensitivity is particularly important to reduce the risk of missed diagnoses. Our model achieved a sensitivity of 98.0%, which is higher than that reported in the large-scale deep learning study by Han et al. (92.6%) and the global meta-analysis by Tan et al. (94.3%). While previous studies have often observed a tradeoff between sensitivity and specificity, such as the work by Cho et al., who reported a specificity of 64.7%, our model achieved a specificity of 88.9%. This suggests that the proposed approach may provide improved discrimination between mild OSA patients and healthy individuals while limiting excessive false alarms.

At the moderate threshold, the model demonstrated a relatively balanced performance, with a sensitivity of 89.5% and a specificity of 90.9%. These results are comparable to those reported by Han et al. (sensitivity 90.9%, specificity 94.4%) and show improved sensitivity compared with the results reported by Wang et al. (83.5%) and Xie et al. (84.7%).

For the identification of severe OSA, the proposed framework showed stable performance. While some existing methods reported reduced sensitivity in severe cases, for example, Xie et al., who reported a sensitivity of 58.1%, our model achieved a sensitivity of 89.3%. In addition, a specificity of 100% was obtained for severe cases. Although this result should be interpreted with caution, it indicates that the model did not misclassify non severe subjects as severe OSA in this dataset, which may be beneficial for clinical decision support.

### 4.5. Limitations

While our framework demonstrates robust screening performance, several limitations warrant acknowledgement:

Sample Size and Demographics: Although the study validated the model across two distinct centers, the total sample size remains relatively modest. Furthermore, the cohort consists exclusively of adults. Since the pathophysiology and acoustic characteristics of pediatric OSA may differ significantly from those of adults, our current findings cannot be directly extrapolated to pediatric populations without separate validation.

Impact of Data Imbalance on Severity Classification A notable limitation arises from the high prevalence of severe OSA within the training dataset derived from the Sismanoglio cohort. As observed in the external validation, the model achieves high sensitivity for severe cases, yet its performance variability at lower severity thresholds suggests a bias toward the dominant class. This phenomenon, known as spectrum bias, represents a common challenge in clinical AI. Models trained on tertiary care cohorts with high disease prevalence often demonstrate superior performance on severe cases while exhibiting reduced accuracy for borderline mild or moderate presentations. To mitigate this skew, future work must prioritize training on larger, community-based cohorts that feature a balanced distribution of AHI severity.

Recording Equipment and Real-world Deployment: Both datasets utilized high-fidelity professional microphones (e.g., Behringer ECM8000, Sony PCM-D10) in controlled hospital environments. We have not yet quantified the performance degradation that might occur when using consumer-grade hardware. Deploying this algorithm on portable devices (e.g., smartphones) introduces specific challenges, including variable microphone frequency responses, automatic gain control (AGC) artifacts, and aggressive audio compression algorithms, all of which could distort the spectral features of breathing sounds. Additionally, uncontrolled home environments introduce high-interference acoustic scenarios, such as loud snoring from a bed partner, television noise, or air conditioning sounds, that were not fully represented in our clinical recordings.

Inherent Acoustic Limits and Emerging Clinical Metrics As detailed in the section on Challenges in Hypopnea Detection and Clinical Implications, relying solely on audio signals presents inherent limitations due to the acoustic ambiguity of hypopnea events and the absence of multimodal physiological confirmation. Furthermore, this lack of multimodal data restricts the assessment of emerging clinical metrics that are increasingly critical for patient prognosis. Indicators such as hypoxic burden [38] and respiratory event-related pulse rate response [39] have gained significant attention for their ability to predict cardiovascular risks associated with OSA. However, a single-modality audio framework is fundamentally incapable of measuring these physiological consequences, limiting its utility in screening for patients at high risk of comorbidities. Consequently, future research should investigate multi-modal fusion approaches, such as integrating audio analysis with data from wearable pulse oximeters, to capture these physiological dimensions and provide a more reliable, multi-dimensional screening outcome.

### 4.6. Future Directions

Edge Deployment on Consumer Devices: We aim to leverage the lightweight nature of our CRNN architecture to deploy the model directly on smartphones for edge computing. This on-device processing approach not only enables real-time monitoring without internet dependency but also ensures user data privacy by eliminating the need to upload sensitive audio recordings to the cloud.

Wearable-Integrated Multi-modal Fusion: To overcome the acoustic limitations in detecting hypopnea, we plan to investigate multi-modal fusion by integrating audio analysis with data from consumer wearables (e.g., smartwatches). Specifically, combining audio signals with SpO2 and ECG/PPG data could significantly enhance the recognition accuracy of subtle respiratory events. Furthermore, wearable devices can provide a precise estimation of Total Sleep Time rather than Total Recording Time, allowing for a much more accurate calculation of the AHI in home settings.

High-Standard Database Construction: To address the limitations of current public datasets, future efforts must focus on establishing a large-scale, high-quality database derived from community-based populations. This initiative should prioritize a balanced distribution of AHI severity and respiratory event phenotypes, ensuring that hypopnea events are sufficiently represented to prevent the masking effect where high apnea counts obscure poor hypopnea detection. To guarantee the reliability of the gold standard, Polysomnography annotations should be performed independently by two certified technicians with formal evaluation of inter-scorer agreement. Additionally, collecting audio data directly from uncontrolled home environments is essential to validate model robustness against real-world acoustic interference.

## 5. Conclusions

This study presents a fine grained and lightweight dual stream CRNN framework that integrates log Mel spectrograms with frame level energy profiles. The proposed architecture enables accurate identification of respiratory events and shows improved performance in detecting hypopnea, which remains a challenging task due to its subtle acoustic characteristics and its similarity to normal breathing patterns.

Rigorous validation across two independent cohorts suggests that the model demonstrates good generalization capability. The results indicate that the proposed framework maintains a high level of agreement with standard PSG in both temporal localization of respiratory events and patient level AHI estimation across different recording environments. In the external validation cohort, the model showed favorable screening performance, particularly in identifying severe cases, while maintaining high sensitivity for mild OSA screening.

Overall, these findings suggest that the proposed framework may provide a reliable and clinically interpretable solution for non-contact OSA screening. In addition, its computational efficiency supports the potential for future deployment in long-term home sleep monitoring applications on mobile and edge devices.

## Figures and Tables

**Table 1:** Frame-level classification performance of the proposed model on the internal test set.

Class	Precision	Recall	F1-Score
Normal	0.92	0.87	0.89
Hypopnea	0.25	0.41	0.31
Apnea	0.74	0.71	0.72
Macro Average	0.63	0.66	0.64
Overall Accuracy	—	—	0.81

**Table 2:** Ablation study investigating the impact of the BiLSTM module and auxiliary Energy stream.

Model Variant	Accuracy	Macro Recall	Macro F1
Dual-Stream CRNN	0.81	0.66	0.64
*w*/*o* Energy Stream	0.79	0.64	0.62
*w*/*o* BiLSTM Module	0.65	0.37	0.36

**Table 3:** Diagnostic screening performance of the proposed model across standard clinical AHI thresholds on the external set.

Threshold	Acc (%)	Sens (%)	Spec (%)	PPV (%)	NPV (%)	Kappa	AUC
AHI ≥ 5	96.67	98.04	88.89	98.04	88.89	0.869	0.97
AHI ≥ 15	90.00	89.47	90.91	94.44	83.33	0.789	0.97
AHI ≥ 30	95.00	89.29	100.00	100.00	91.43	0.899	0.99

Abbreviations: AHI: Apnea-Hypopnea Index; Acc: Accuracy; Sens: Sensitivity; Spec: Specificity; PPV: Positive Predictive Value; NPV: Negative Predictive Value; AUC: Area Under the Receiver Operating Characteristic Curve.

**Table 4:** Performance comparison with existing non-contact audio-based methods for OSA screening.

First Author	Year	Country	Sample Size	AHI ≥ 5		AHI ≥ 15		AHI ≥ 30	
				Sn (%)	Sp (%)	Sn (%)	Sp (%)	Sn (%)	Sp (%)
Cho [18]	2022	South Korea	423	90.8	64.7	87.3	70.6	83.0	80.3
Wang [21]	2022	China	135	93.6	83.3	83.5	95.8	95.6	91.6
Romero [22]	2022	UK	103	NR	NR	79.0	80.0	78.0	93.0
Le [23]	2023	South Korea	1315	97.0	89.0	85.0	84.0	96.0	91.0
Xie [28]	2023	Netherlands	172	97.7	50.0	84.7	75.0	58.1	96.5
Han [37]	2024	South Korea	1416	92.6	84.3	90.9	94.4	93.3	94.4
Tan [26]	2025	Global (Meta)	7234	94.3	78.5	86.3	87.3	86.3	89.5
Our Model	2025	China/Greece	346	98.0	88.9	89.5	90.9	89.3	100.0

Abbreviations: AHI: Apnea-Hypopnea Index; Sn: Sensitivity; Sp: Specificity.

## Data Availability

The data presented in this study are available on request from the corresponding author due to privacy and ethical restrictions, as the dataset contains sensitive clinical information involving human participants.

## Supplementary Materials

The following supporting information can be downloaded at: https://www.mdpi.com/article/10.3390/diagnostics16040577/s1, Table S1: Implementation Hyperparameters and Training Settings; Table S2: Comparative analysis of detection performance and edge efficiency metrics across varying input window durations; Table S3: Sensitivity analysis of post-processing parameters.
