# Supplementary material for: Fine-Grained and Lightweight OSA Detection: A CRNN-Based Model for Precise Temporal Localization of Respiratory Events in Sleep Audio
Source: Diagnostics (Basel). 2026 Feb 14;16(4):577. doi: 10.3390/diagnostics16040577 (PMC12939127; doi:10.3390/diagnostics16040577)
Supplement: Supplementary file 1 [file diagnostics-16-00577-s001.zip › diagnostics-4117382-supplementary.pdf]

Article Title: Fine-Grained and Lightweight OSA Detection: A CRNN-Based Model for Precise Temporal Localization of Respiratory Events in Sleep Audio

Journal Name: Diagnostics

Author information:

Demin Han (Corresponding author):

Affiliations: A, B, C

E-mail: deminhan_ent@hotmail.com

Yanru Li (Corresponding author):

Affiliations: A, B, C

E-mail: liyanruru@aliyun.com

Mengyu Xu (First Author):

Affiliations: A, B

E-mail: ent_xu@ccmu.edu.cn

A: Department of Sleep Medical Center, Beijing Tongren Hospital, Capital Medical University, Beijing 100730, China

B: Key Laboratory of Otorhinolaryngology Head and Neck Surgery, Ministry of Education, Capital Medical University, Beijing 100730, China

C: Xinjiang Key Laboratory of Biopharmaceuticals and Medical Devices, Xinjiang Medical University, Urumqi 830054, China

Supplementary Table S1. Implementation Hyperparameters and Training Settings

Parameter	Value / Configuration
Random Seed	42
Early Stopping Patience	5 Epochs
Batch Size	24
Maximum Epochs	30
Optimizer	AdamW
Initial Learning Rate	5×10^{-4}
Weight Decay	5×10^{-2}
LR Scheduler	ReduceLROnPlateau (Factor=0.5, Patience=2)
Gradient Clipping Norm	1.0
Dropout Rates	0.2 (BiLSTM layers), 0.1 (Classification Head)
Loss Type	Hybrid Event Loss (Focal + Dice)
Focal Loss Parameters	γ=2.0, weights α= [1.0, 4.0, 2.0] (Normal, Hypopnea, Apnea)
Dice Loss Weight	0.5
SpecAugment	Freq Mask (F=15), Time Mask (T=40)
Signal Gain	Random factor U (0.8, 1.2)

Supplementary Table S2. Comparative analysis of detection performance and edge efficiency metrics across varying input window durations

Window Duration	Accuracy	Macro Recall	Macro F1-Score	Params (M)	GFLOPs/sec	GPU Latency (ms)	CPU Latency (ms)
5 seconds	0.75	0.49	0.49	1.73	0.40	2.19	7.03
30 seconds	0.79	0.61	0.60	1.73	0.40	5.50	38.72
**60 seconds**	0.81	0.66	0.64	1.73	0.40	11.60	84.81
90 seconds	0.82	0.66	0.64	1.73	0.40	17.09	128.97
120 seconds	0.81	0.65	0.63	1.73	0.40	22.57	173.48

Notes: GPU Latency: Inference latency was measured on an NVIDIA GeForce RTX 5070 Ti Laptop GPU with a batch size of 1. CPU Latency: Inference latency was measured on an AMD Ryzen 9 8945HX Processor with a batch size of 1.

Abbreviations: Params (M): Model parameters in millions; GFLOPs/sec: Giga Floating Point Operations per second (representing computational density).

Supplementary Table S3. Sensitivity analysis of post-processing parameters

Parameter	Setting	Average IoU	AHI Correlation (r)	AHI MAE (events/h)
Median Filter	Size = 3	0.819	0.959	4.80
	**Size = 5**	0.820	0.960	4.79
	Size = 10	0.817	0.957	4.82
Gap-Merging	3 seconds	0.821	0.959	4.80
	**5 seconds**	0.820	0.960	4.79
	8 seconds	0.802	0.948	5.29
Min Duration	8 seconds	0.818	0.968	4.72
	**10 seconds**	0.820	0.960	4.79
	12 seconds	0.818	0.943	5.60

Notes: The "Size=5", "5 seconds", and "10 seconds" settings (highlighted) represent the final configuration.

Abbreviations: IOU: Intersection over Union; AHI: Apnea-Hypopnea Index; MAE: Mean Absolute Error.
